# Supplementary material for: Detection of Viral −RNA and +RNA Strands in Enterovirus-Infected Cells and Tissues
Source: Microorganisms. 2020 Dec 4;8(12):1928. doi: 10.3390/microorganisms8121928 (PMC7761939; doi:10.3390/microorganisms8121928)
Supplement: Supplementary file 1 [file microorganisms-08-01928-s001.pdf]

Supplementary material for article: Salmikangas et al., 2020. Detection of - and + RNA strands in enterovirus infected cells and tissues.

Supplementary table 1. Antibodies used in IF and bDNA FISH.

Antibody	Type	Final concentration	Origin
CVA9-8863 rabbit monoclonal antibody	Primary (rabbit anti-CVA9 capsid)	1:100	Merja Roivainen, THL, Helsinki
CVA9-861 rabbit monoclonal antibody	Primary (rabbit anti-CVA9 capsid)	1:100	Merja Roivainen, THL, Helsinki
CVA9-K3 rabbit monoclonal antibody	Primary (rabbit anti-CVA9 capsid)	1:100	Merja Roivainen, THL, Helsinki
J-2 mouse mono-clonal antibody	Primary (mouse anti-dsRNA)	1:8000	Scicons (Prod.No. 10010200)
Goat anti-Rabbit IgG (H+L) Secondary Antibody, Alexa Fluor 488 (GAR488)	Secondary (goat anti-rabbit)	1:400	Invitrogen/ Molecular Probes, Ref. A-11008
Goat anti-Mouse IgG (H+L) Secondary Antibody, Alexa Fluor 488 (GAM488)	Secondary (goat anti-mouse)	1:400	Invitrogen/ Molecular Probes, Ref. A-11029
Goat anti-Mouse IgG (H+L) Secondary Antibody, Alexa Fluor 633 (GAM633)	Secondary (goat anti-mouse)	1:400	Invitrogen/ Molecular Probes, Ref. A-21052

Supplementary table 2. ViewRNA in-house/custom probe set sequences used for +RNA (EVAB+) and -RNA (EVAB-) detection. Probe set sequence information of EVAB+ has previously been published in [24].

Oligo number	EVAB+	EVAB-
1	gactcatggatctccttcattgg	ccaatgaaggagatccatgagtc
2	gcgtccttagtccaacgaatg	cattcgttggactaaggacgc
3	cataccctgagtaatcaaaagcaa	ttgcttttgattactcagggtatg
4	aaccaaacaggactaagactagcat	atgctagtcttagtcctgtttggtt
5	taaggtaaactgagtcatttaggctac	gtagcctaaatgactcagtttacctta
6	acaggtggccaaaagtcattc	gaatgacttttggccacctgt
7	tgtacatcatggttctgtgtgtagg	cctacacacagaaccatgatgtaca
8	tcctgctttggttgggaaat	atttcccaaccaaagcagga
9	gattttggcagagatcatccataa	ttatggatgatctctgccaaaatc
10	aatagtgacatgtctttcccatctg	cagatgggaaagacatgtcactatt
11	tctggaggtagtgagtacacactgg	ccagtgtgtactcactacctccaga
12	tgtacccatcaaagtgatctggg	cccagatcactttgatgggtaca
13	tatgtctttcatgggcatgaca	tgtcatgcccatgaaagacata
14	ttggtccatctgattgattcatg	catgaatcaatcagatggaccaa
15	gatcttgggtgttctttggatct	agatccaaagaacacccaagatc

16	taggcacagtgagcgcacat	atgtgcgctcactgtgccta
17	ccttgtacaccttcagcattagtg	cactaatgctgaaggtgtacaagg
18	cctgaattgatccaagtcaatcc	ggattgacttggatcaattcagg
19	cacatcatcaccatatgcaatcat	atgattgcatatggtgatgatgtg
20	ggccatgggtatgatgcaat	attgcatcatacccatggcc
21	ctggtgccagaacaccctg	cagggtgttctggcaccag
22	tgttgttgatcattgagttgaagatg	catcttcaactcaatgatcaacaaca
23	ttgcacaggtagtcaatgtagtttg	caaactacattgactacctgtgcaa
24	tgtctctgtacaggtggtgggag	ctcccaccacctgtacagagaca
25	cagaccatacttgtccatgcact	agtgcatggacaagtatggtctg
26	tcacataagtcaccattggtaggtt	aacctaccaatggtgacttatgtga
27	ccttgttcatcattgaagtagtgct	agcactacttcaatgatgaacaagg
28	gagctctcaatgaactcgatttct	agaaatcgagttcattgagagctc
29	ggtgtgccacccaggttca	tgaacctgggtggcacacc
30	ttgtacatgagcattctcttggtg	caccaagagaatgctcatgtacaa
31	gctagcacagcctcattgacct	aggtcaatgaggctgtgctagc
32	ggggaacttgctggtgttaatt	aattaacaccagcaagttcccc
33	tgtcaaagtggaatctccttgct	agcaaggagattccactttgaca
34	tgtacatggaaataacttcaatgttca	tgaacattgaagttatttccatgtaca
35	ctctctttgcctcagctgcat	atgcagctgaggcaaagagag
36	tcatcttcttctcaagggagaaca	tgttctcccttgagaagaagatga
37	tcagtgaacttcttgagccacc	ggtggctcaagaagttcactga
38	ccttgcaggcattggtcatc	gatgaccaatgcctgcaagg
39	tgatggctatccactccatgc	gcatggagtggatagccatca
40	agccactcaataaatttctgaattt	aaattcagaaatttattgagtggct
41	cacactacccactggtttgtgg	ccacaaaccagtgggtagtgtg
42	gcagagttgcccgttacga	tcgtaacgggcaactctgc
43	ccaaagtagtcggttccgct	agcggaaccgactactttgg
44	aagaataaaaggaaacacggacac	gtgtccgtgtttccttttattctt
45	ttgtcaccataagcagccaatat	atattggctgcttatggtgacaa
46	caatagctatatggtaacaatctttcaa	ttgaaagattgttaccatatagctattg
47	ggggaaacagaagtgcttgat	atcaagcacttctgtttcccc
48	ccaacgcagccaccgc	gcggtggctgcgttgg
49	cccatgggcaggccg	cggcctgcccatggg
50	gagcgtcccatgggttgc	gcaacccatgggacgctc
51	ctcttcgcaccatgtcagtattg	caatactgacatggtgcgaagag
52	gtgagcagtctattgatactcagtcc	ggactgagtatcaatagactgctcac
53	acgttttctccttcaaccgc	gcggttgaaggagaaaacgt
54	agtagttggccgggtaacga	tcgttacccggccaactact
55	tcatggtgttactaggtttctcga	tcgagaaacctagtaacaccatga
56	agcgaaacactccgcaactt	aagttgcggagtgtttcgct
57	cctgatctacactggggtgctg	cagcaccccagtgtagatcagg
58	ggaatgcggtgactcatcga	tcgatgagtcaccgcattcc
59	cacggtcgcccgtgg	ccacgggcgaccgtg
60	ccagagtgctagcgcccagt	actgggcgctagcactctgg
61	gcgcacaaaggtaccgtgata	tatcacggtacctttgtgcgc

Supplementary table 3. Components of the RT reaction.

Reagent	Amount (µl) per sample	Final concentration	Origin
Nuclease free water	9.0	-	Alfa Aesar (Ref. J71786)
5x RT-buffer	8.0	1x	Promega (Ref. M5301)
2.5 mM dNTP mix with dATP, dTTP, dCTP and dGTP	8.0	0.5 mM each	Promega (Ref. U1330)
10 µM ENRI (3+ or 4-) primer	4.8	1.2 µM	Thermo Scientific Custom Standard DNA Oligos
RNase inhibitor	0.1	4 units	Promega (Ref. N2518)
RT-enzyme (M-MLV RT [H–])	0.1	20 units	Promega (Ref. M530A)
RNA template	10.0	-	Viral RNA extraction
Total	40.0	-	-

Supplementary table 4. Components of the qPCR reaction.

Reagent	Amount (µl) per sample	Final concentration	Origin
Nuclease free water	4.5	-	Alfa Aesar (Ref. J71786)
iQ SYBR Green Supermix	12.5	1x	Bio-Rad (Ref. 1708886)
10 µM ENRI 4- primer	1.5	0.6 µM	Thermo Scientific Custom Standard DNA Oligos
10 µM ENRI 3+ primer	1.5	0.6 µM	Thermo Scientific Custom Standard DNA Oligos
cDNA template	5.0	1:500	RT-reaction
Total	25.0	-	-

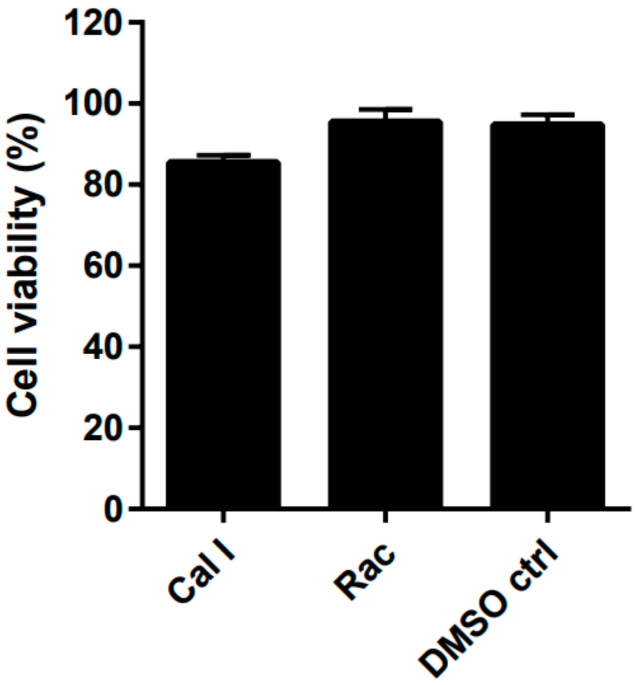

Supplementary figure 1. Cell viability assay for the antiviral drugs Cal-I1 and Rac1-I. After a 5-hour incubation, the antiviral drug Cal-I1 lowers cell viability by ~10%, and the antiviral drug Rac1-I doesn't lower cell viability, compared to a DMSO control.

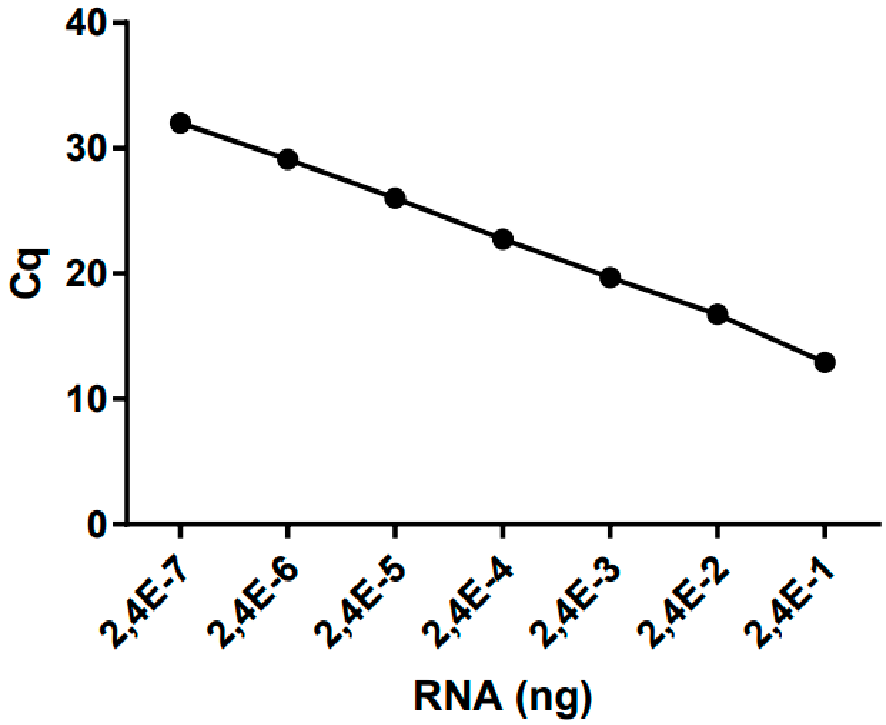

Supplementary figure 2. A concentration series of RNA isolated from purified CVA9 and detected by qPCR. 10-fold dilutions were used, triplicate samples were measured and the average Cq-values from the triplicates were plotted against the RNA amounts.
